# Revealing culturable fungal microbiome communities from the Arabian Peninsula desert representing a unique source of biochemicals for drug discovery and biotechnology

**DOI:** 10.12688/f1000research.158130.1

**Published:** 2024-12-17

**Authors:** Walaa Mousa, Najwa Alramadan, Rose Ghemrawi, Tareq Abu Izneid

**Affiliations:** 1 College of Pharmacy, Mansoura University, Mansoura, 35516, Egypt; 2 College of Pharmacy, Al Ain University, Al Ain, Abu Dhabi, 64141, United Arab Emirates; 3 AAU Health and Biomedical Research Center, Al Ain University, Al Ain, Abu Dhabi, 112612, United Arab Emirates; 4 Monash Rural Health, Churchill, School of Rural Health, Faculty of Medicine, nursing and health sciences, Monash University, Victoria, 3844, Australia

**Keywords:** Desert microbiome, culturable fungi, bioactive molecules, antioxidant, antimicrobial, and cytotoxic.

## Abstract

**Background:**

Microbes living at extremes evolve unique survival strategies to adapt to challenging environmental conditions. Among these strategies is their distinctive metabolic potential and ability to produce specialized metabolites enabling them to compete for limited resources and defend against predators. These metabolites have significant potential in pharmaceutical and industrial applications, particularly in the development of drugs and biochemicals.

**Objectives:**

This study aimed to investigate the culturable fungal communities associated with four desert plants and their surrounding soils in the Arabian Peninsula desert to identify their bioactive properties.

**Methods:**

A total of 12 distinct fungal species were isolated from the plants and soils. Each plant hosted a unique set of fungi, demonstrating the diversity of desert-adapted fungal communities. Biological activities of the fungal extracts were evaluated through various assays, including antimicrobial, antifungal, anticancer, and antioxidant properties.

**Results:**

*Panicum turgidum* harbors the most diverse fungal community, dominated by genera such as *Mucor*, *Aspergillus*, *Colletotrichum*, *Alternaria*, and *Chaetomium.* *Aspergillus* species comprise 33% of the total isolates, followed by *Fusarium* at 16%. All extracts exhibit diverse activities, with *Aspergillus* species demonstrating the highest antioxidant activities and total phenolic and flavonoid content. Fungi from *P. turgidum*, particularly *Mucor* sp., *Aspergillus* sp., and *Curvularia* sp., display potent activity against *Staphylococcus aureus*, while *Mucor* sp., *Chaetomium* sp., and *Curvularia* sp. exhibit moderate inhibition against *Pseudomonas aeruginosa.*

**Conclusion:**

This study highlights the importance of exploring extremophilic microorganisms, such as those found in desert ecosystems, as they offer a wealth of compounds that could address current challenges in drug discovery and biotechnology.

## Introduction

Microbes thrive in extreme environments, such as deserts, arctics, and deep-sea ecosystems by evolving unique survival mechanisms adapting to harsh conditions. These adaptations often include the production of specialized metabolites that play essential roles in competition, defense, and stress tolerance. For example, *Penicillium chrysogenum*, initially isolated from desert soil, produces a variety of antimicrobial compounds, including the β-lactam antibiotic penicillin ^1^ Similarly, *Aspergillus terreus* produces lovastatin, a statin used to lower cholesterol, ^2^ while *Streptomyces hygroscopicus*, which has been isolated from alkaline soils, secretes rapamycin, a potent immunosuppressant. ^3^ Beyond their pharmaceutical potential, fungi are also exploited at an industrial level for the production of various compounds. For example, *Aspergillus niger* produces over 70% of global citric acid demand, ^4^ while *Trichoderma reesei* produces cellulases crucial for biofuel production and textile processing. ^5^ *Fusarium venenatum* is used to produce mycoprotein, a sustainable meat substitute, ^6^ and *Aspergillus oryzae* is essential in fermenting traditional Asian foods such as soy sauce and miso. ^7^ These examples showcase the potential of fungal metabolites across various pharmaceuticals and industrial sectors. Among challenging habitats is the arid desert, with extreme temperatures, UV radiation, and limited nutrients. These conditions drive the evolution of novel metabolic pathways in these extremophilic organisms, representing a largely untapped source of bioactive compounds with potential in drug discovery and biotechnology. In this study, we aimed to characterize the culturable fungal communities of four desert plants native to the Arabian Peninsula deserts. These plants included *Panicum turgidum*, *Halocnemum strobilaceum*, *Haloxylon persicum*, and *Arnebia hispidissima.* These plants have not been explored extensively for their fungal microbiomes, despite the essential role these microbes play in enhancing plant resilience and overall health.

*P. turgidum* is a desert xerophyte that thrives in arid regions, including the Arabian Peninsula and parts of North Africa and Asia. This plant is characterized by its extensive drought resistance, with a significant role as a nurse plant in desert ecosystems. ^8^ Its rhizosphere is profoundly colonized by Arbuscular Mycorrhizal Fungi (AMF), establishing a crucial symbiotic relationship that enhances nutrient uptake, water retention, and protection against pathogens. ^9^ Recent studies have shown that AMF inoculation can improve drought tolerance in *Panicum turgidum*, mitigating oxidative stress and promoting chlorophyll production ^10^

*H. strobilaceum* is well-adapted to saline and hypersaline habitats, such as salt marshes and alkali flats, making it native to regions like the Red Sea and Mediterranean. ^11, 12^ Compounds derived from *H. strobilaceum* have demonstrated strong antimicrobial, antioxidant, and antibiofilm activities. For instance, recent research identified two promising compounds, one an alkaloid effective against various pathogens, and the other specifically inhibiting biofilm formation by *Pseudomonas aeruginosa.* ^13^ Furthermore, the ethyl acetate extract of this plant has shown anticancer activity against common cancer cell lines in Egypt, including prostate (PC-3), lung (A-549), and breast (MCF-7) cancer. ^14^ *H. persicum*, another well-known desert xerophyte, survives in arid regions due to its high resilience to drought stress, supported by its production of intrinsic compounds and metabolites. ^15^ Found across western Asia, this plant significantly contributes to soil health and seed bank diversity within desert ecosystems. ^16^ A study highlighted that the rhizospheres of *H. persicum* are rich in archaea and fungi, which play a critical role in nutrient cycling and promoting plant growth under harsh conditions. ^17^ *A. hispidissima* inhabits arid and semi-arid regions in India and northern Africa and is widely distributed in the UAE. This plant is recognized for its medicinal properties, particularly its extract containing shikonin, which exhibits significant anti-cancer properties through mechanisms targeting cancer cell death. ^18^ Additionally, it has been traditionally used in Indian medicine for treating various infections due to its potent antimicrobial properties. ^19^ The successful biosynthesis of silver nanoparticles using root extracts of *A. hispidissima* has been reported, showcasing their potent antioxidant and antimicrobial activities against several pathogens. ^20^

This study aims to explore the fungal communities associated with these resilient desert plants, identifying fungal species and their biological significance for potential applications in pharmaceuticals and biotechnology.

## Methods

### Collection of plant samples

In a prior study, ^21^ we explored culturable bacterial communities from both the rhizosphere (R) and endosphere (E) of four native desert plants of the Arabian Peninsula: *Halocnemum strobilaceum* (HS), *Panicum turgidum* (PT), *Haloxylon persicum* (HP), and *Arnebia hispidissima* (AH). This study extends those findings by isolating and evaluating fungal communities associated with these plants. The methodologies for plant collection and sample preservation have been detailed previously. ^21, 22^ In brief, five replicates from each plant were gathered from different sites near the UAE, which varied in soil properties. Collected samples, comprising both roots and surrounding rhizosphere soils, were obtained in October 2022, during which daytime temperatures ranged between 34 and 40 °C with minimal rainfall.

### Fungal epiphyte isolation

A modified root-washing technique, based on the method published by Banno et al, ^23^ was employed to isolate epiphytic fungi. Briefly, the roots were immersed in sterile water and shaken at 250 rpm for 30 minutes, a step that was repeated three times. Root washes were pooled, serially diluted (up to 10 ^5^), and 200 μL of each dilution was spread on three types of media: potato dextrose agar (PDA, HiMedia, India # MH096), Sabouraud dextrose agar (SDA, HiMedia, India # MV063), and yeast maltose agar (YMA, HiMedia, India # M1967). All media have been prepared according to the manufacturer protocol. To prevent bacterial growth, all media were supplemented with chloramphenicol (200 μg/L). Plates were incubated at 25 °C for 5 days. Fungal colonies, selected based on their morphology, were subcultured multiple times (3–5 repetitions) until pure strains were obtained. For long-term storage, fungal spores and mycelia were suspended in 25% glycerol and stored at -20 °C.

### Fungal endophyte isolation

The protocol for isolating endophytic fungi involved surface-sterilizing the root tissues, followed by similar culturing techniques as for epiphytes. Root surface sterilization was conducted by first sonication in autoclaved water for 5 minutes to remove soil particles, followed by a series of ethanol (95% for 3 minutes) and sodium hypochlorite (3% NaOCl for 5 minutes) treatments. Between these steps, the roots were thoroughly rinsed with sterile water. Sterilization success was confirmed by rolling sterilized roots on PDA plates and incubating them at 25 °C and 37 °C; no microbial growth was observed. The sterilized roots were then sectioned and ground in a sterile mortar, after which the tissue was plated on PDA, SDA, and YMA media. Subculturing and maintenance followed the same protocol as for the epiphytic fungi.

### Molecular identification of fungal isolates

DNA extraction from fungal isolates was performed using a commercial DNA extraction kit (Norgen Bioteck, Canada). Briefly, the fungi were cultured in PD broth for 3 days, after which the mycelia were collected and ground in a sterile mortar. DNA extraction proceeded according to the manufacturer’s instructions, and the quality and quantity of DNA were assessed using gel electrophoresis and a nanodrop spectrophotometer, respectively. Taxonomic identification was carried out using ITS primers: ITS1 (5′-TCCGTAGGTGAACCTGCGG-3′) and ITS4 (5′-TCCTCCGCTTATTGATATGC-3′). PCR amplification involved an initial denaturation at 95°C for 5 minutes, followed by 35 cycles of 1-minute denaturation at 94°C, 30-second annealing at 55°C, and a 2-minute extension at 72°C, concluding with a final 10-minute extension at 72°C. PCR products were purified with the QIAquick gel purification kit (Qiagen, Germany) and sequenced. Sequences were aligned using BLAST against the NCBI database and deposited in.

### Biological assays

All fungal isolates were cultured in 3 L Fernbach flasks containing PDA medium and incubated at 28 °C for 10 days. After this period, the culture was extracted by shaking with ethyl acetate (3 times the volume), and this extraction was repeated three times. The extract was then filtered and concentrated to dryness using a rotary evaporator (BUCHI R100, Schweiz). The resulting residue was dissolved in DMSO at a stock concentration of 100 μg/μL, which was subsequently used to evaluate various biological activities.

### Antioxidant activities

To determine the antioxidant activity of the fungal extracts, we evaluated their free radical scavenging capacity colorimetrically using DPPH as a source of free radicals. A freshly prepared DPPH solution (50 μg/mL) was combined with a serial dilution of each fungal extract (ranging from 100 to 1 μg/mL), shaken, and allowed to incubate in the dark for 30 minutes at 20 °C. The absorbance was recorded at 517 nm using a spectrophotometer (OmegaStar, Germany). The percentage of radical scavenging activity was calculated using the formula:

Radical Scavenging Activity(%)=[1−(Abs(517 nm)of the sample/Abs(517 nm)of the control)]×100.

### Total phenolic content

The total phenolic content was determined using the Folin-Ciocalteu method. ^23^ In brief, 1 μL of the extract (100 μg/μL) was combined with 100 μL of the Folin-Ciocalteu reagent (10% v/v) and incubated for 15 minutes. Following this, 2 μL of sodium carbonate (7%, w/v) was added to the mixture to neutralize it. The samples were then stored in the dark for 2 hours. Absorbance was recorded at 765 nm using a spectrophotometer. A calibration curve was established with gallic acid concentrations ranging from 5 to 200 μg/mL, and total phenolic content was expressed as micrograms of gallic acid equivalents (μg GAE) per milligram of EA extract.

### Total flavonoid content

The total flavonoid content was evaluated using the aluminum chloride colorimetric method. ^24^ Briefly, 5 μL of the extract (100 μg/μL) was combined with 100 μL of aluminum chloride (3% w/v) and 100 μL of potassium acetate (1 M). The samples were incubated at 25°C for 30 minutes. The absorbance was then measured at 420 nm, with the solvent used for dissolving the extract serving as the blank control. Quercetin (5–200 μg/mL) was utilized to generate the calibration curve. The results for total flavonoid content were reported as micrograms of quercetin equivalents (μg QE) per milligram of EA extract.

### Antimicrobial activities of the extracts

To investigate the antimicrobial properties of the fungal extracts, we employed an agar diffusion assay against human and plant pathogens. The bacterial pathogens included Gram positive indicator Staphylococcus aureus (ATCC 25923) and *Pseudomonas aeruginosa* (BAA-1744). The fungal pathogens included in this study are *C. albicans* (ATCC 18804) and *Fusarium gramineraum* (MYA-4620). Each pathogen was cultured under optimal conditions for growth. We first conducted agar diffusion method and positive extracts were assessed for their minimum inhibitory concentration (MIC) using broth dilution method. For agar diffusion test, a 100 μL of an overnight culture of each pathogen was evenly spread onto agar plates (utilizing media appropriate for each pathogen’s growth). Sterile glass pipettes were then used to create wells in the agar, and 20 μL of 10 μg/μL of each extract was introduced into these wells and the plates were incubated aerobically at 37 °C for 24 hours. After incubation, the plates were examined for zones of inhibition. Positive cultures were processed to determine the MIC. To measure MIC, a single colony of each pathogen was initially cultured for 24 hours in its specific broth medium and subsequently diluted in the same medium at a 1:10,000 ratio, following McFarland Standards. Thereafter, 196 μL of the pathogen suspension was added to each well of a microplate, followed by the addition of 4 μL of serially diluted fungal extracts. Positive controls included amoxicillin (5 μM), and ciprofloxacin (2 μM), and amphotericin B (10 μM). The plates were incubated for 24 hours at the optimum growth condition for each pathogen, after which the optical density at 600 nm (OD600) was recorded using a microplate reader. All concentrations were tested in triplicate, and the experiment was independently replicated. The percentage of growth inhibition was calculated as previously described. ^25^ To determine the minimum bactericidal or fungicidal concentration (MBC) of the fungal extracts, 10 μL of the inhibited samples from the MIC assay (where no growth was observed) were plated onto suitable agar plate and incubated at the best growth condition for each pathogen then inspected for colony formation. Control groups included samples treated with antibiotic or antifungal compound and untreated cultures.

### Cytotoxicity assay

Two human lung cancer cell lines, A549 and H292, were utilized to evaluate the cytotoxic properties of the fungal extracts. The cells were cultured in RPMI medium supplemented with 10% FBS, 1% penicillin-streptomycin solution, and incubated at 37 °C in a 5% CO2 environment. The in vitro cytotoxic activity of the extracts was assessed against the A549 and H292 cells by measuring the formation of insoluble formazan salt, which occurs via the reduction of 3-(4,5-dimethylthiazol-2-yl)-2,5-diphenyl tetrazolium bromide (MTT) by NAD(P)H-dependent cellular oxidoreductase enzymes, directly correlating to the number of viable cells remaining after extract treatment. Tumor cells (30 × 10 ^3^ cells per well) were seeded into 36-well culture plates and incubated for 24 hours at 37 °C in a humidified CO _2_ incubator. After this incubation, the cells were treated with 10 μg/mL of each extract for 24 hours. Control groups included cells treated with 0.1% DMSO and untreated cells. Following treatment, 20 μL of MTT solution was added to each well, and the plates were incubated for an additional 4 hours at 37 °C in a 5% CO _2_ incubator. After incubation, the plates were centrifuged for 20 minutes at 3000 rpm, and the formazan crystals formed were dissolved using 100 μL of DMSO. Absorbance was then measured at 570 nm using a microplate reader.

### Statistical analysis

Data analysis and graphical representation were carried out using one-way ANOVA in GraphPad Prism V9 (GraphPad Software, La Jolla, CA, USA). Similar function could be performed by Microsoft Excel. All experiments were performed in triplicate, and results are presented as means ± standard error of the mean (SEM). Data for statistics are shown in Tables 1-4, together with the raw data. ^101^

## Results

**Figure 1.**
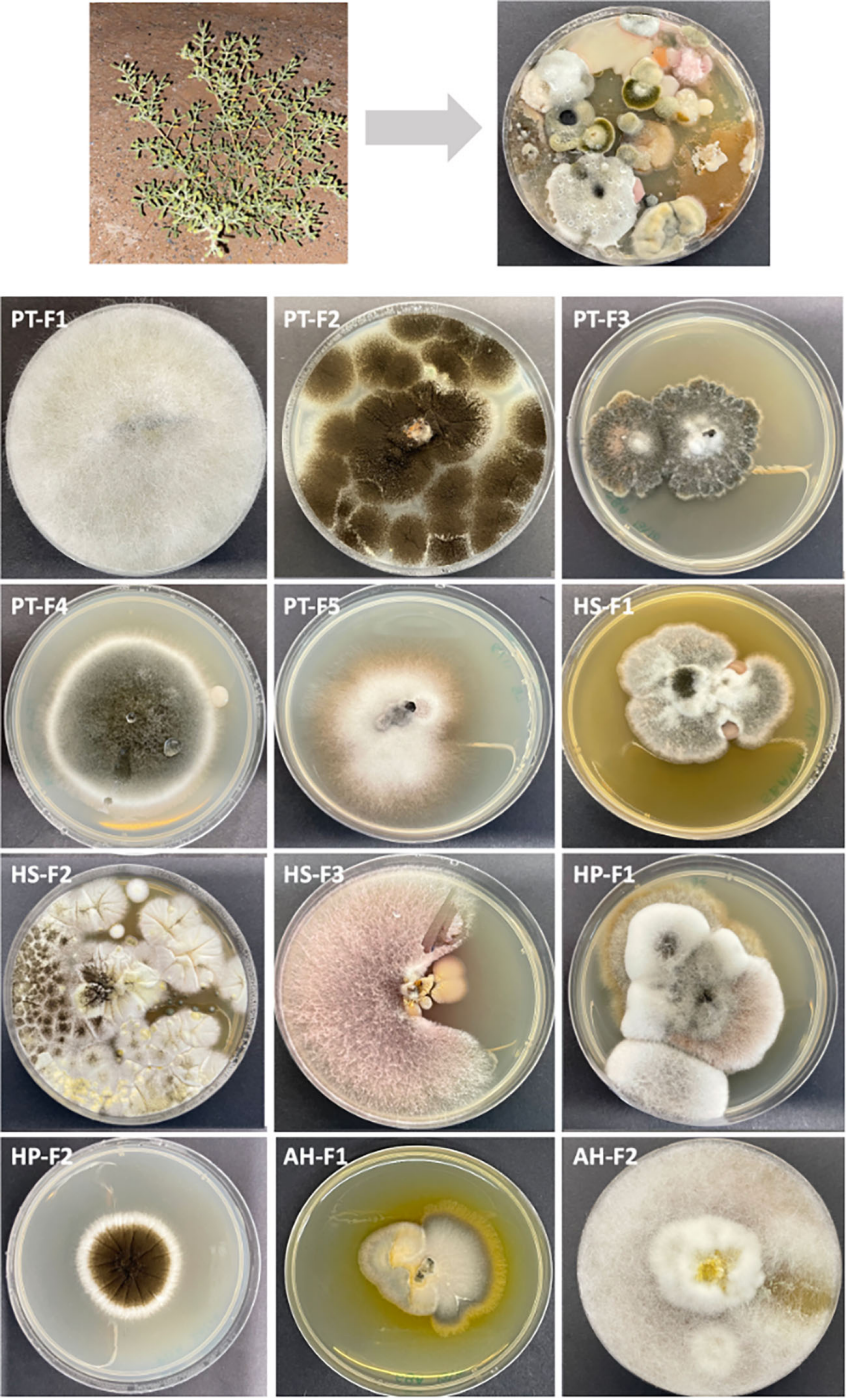
Isolation of culturable fungal species from desert plants. The figure illustrates the process of isolating fungi from plant material and subsequent subculturing to obtain pure isolates.

In this study, we aimed to profile the culturable fungal communities associated with four plants native to the Arabian Peninsula desert. We isolated a total of 12 unique fungal species, identified taxonomically through sequence alignments, and assessed their derived extracts for various biological activities ( Figure 1). From *Panicum turgidum*, we isolated five fungal species identified as *Mucor sp.* (PT-F1), *Aspergillus sp.* (PT-F2), *Colletotrichum sp.* (PT-F3), *Alternaria sp.* (PT-F4), and *Chaetomium sp.* (PT-F5). Additionally, three isolates were obtained from *Halocnemum strobilaceum*, two of which were identified as *Aspergillus* species (HS-F1 and HS-F2), while the other was *Fusarium sp.* (HS-F3). From *Haloxylon persicum*, we identified two unique fungi: *Plectosphaerella sp.* (HP-F1) and *Aspergillus sp.* (HP-F2). Lastly, two fungi were isolated from *Arnebia hispidissima*, namely *Curvularia sp.* (AH-F1) and *Fusarium sp.* (AH-F2). Overall, *Aspergillus* species were the most prevalent, representing 33% of the total recovered fungi across all plant samples.

**Figure 2.**
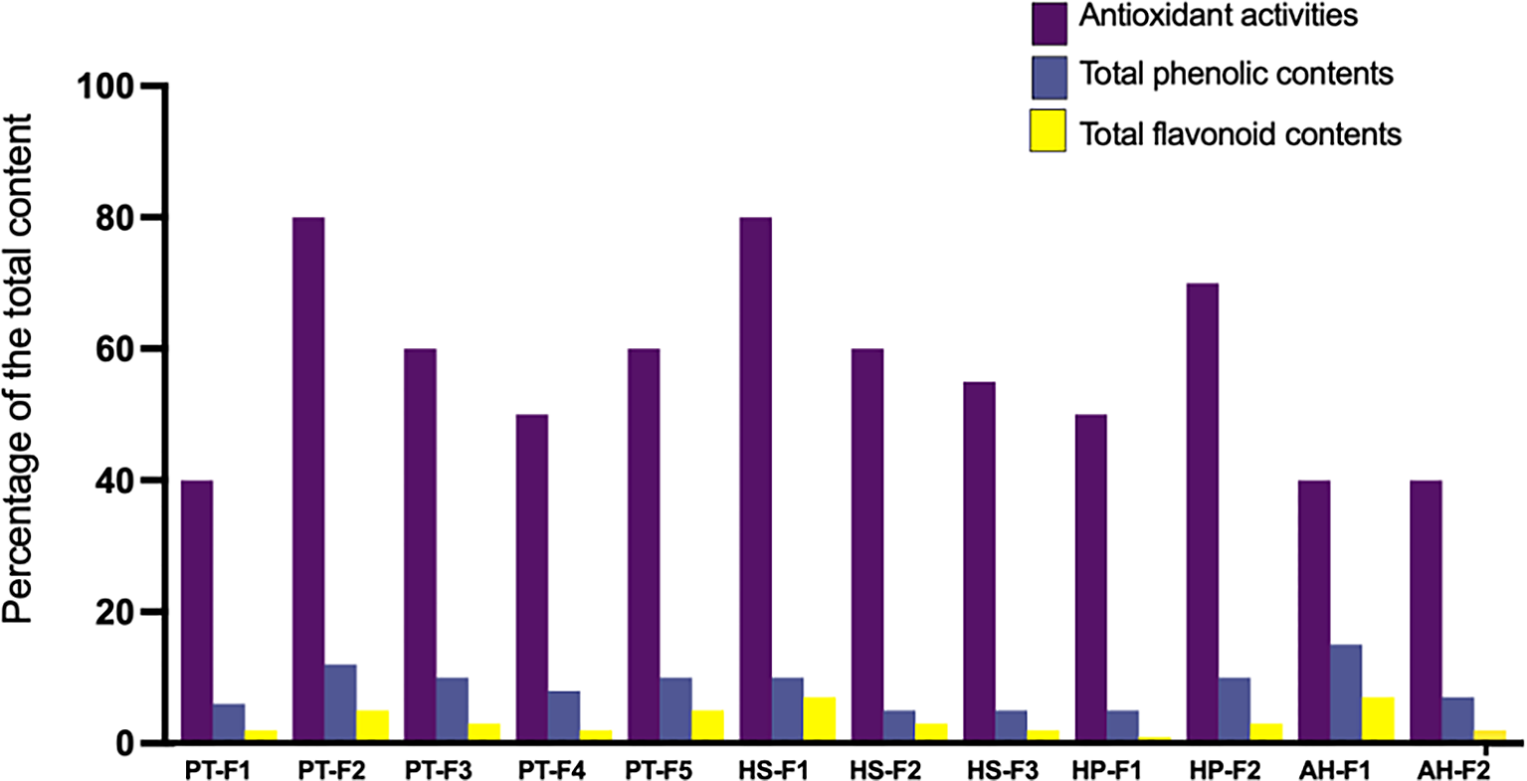
Activities of the crude extracts of cultured fungal species. The graph demonstrates three activities including antioxidant activities measured as % of DPPH reduction, total phenolic content measured as μg of EAG per mg of the extract and total flavonoid contents measured as μg of EQ per mg of the extract. Data are the average of three replicates.

To assess the biological activities of the isolated fungi, we scaled up the fermentation of each species and prepared crude ethyl acetate extracts. These extracts were then subjected to six assays: 1) antioxidant activities, 2) total phenolic content, 3) total flavonoid content, 4) antibacterial assay, 5) antifungal assay, and 6) anticancer cytotoxicity screening. All assessed extracts demonstrated comparable antioxidant activities, with the highest levels observed in *Aspergillus* species PT-F2, HS-F1, and HP-F2 ( Figure 2). In contrast, the lowest antioxidant activities were recorded for PT-F1 ( *Mucor sp.*), AH-F1 ( *Curvularia sp.*), and AH-F2 ( *Fusarium sp.*). Regarding total phenolic content, *Curvularia sp.* exhibited the richest extract, followed by *Aspergillus* species isolated from *Panicum turgidum.* The lowest phenolic content was found in HS-F2 ( *Aspergillus sp.*), HS-F3 ( *Fusarium sp.*), and HP-F1 ( *Plectosphaerella sp.*). The highest flavonoid content was reported for HS-F1 ( *Aspergillus sp.*) and AH-F1 ( *Curvularia sp.*), followed by PT-F2 ( *Aspergillus sp.*) and PT-F5 ( *Chaetomium sp.*).

**Figure 3.**
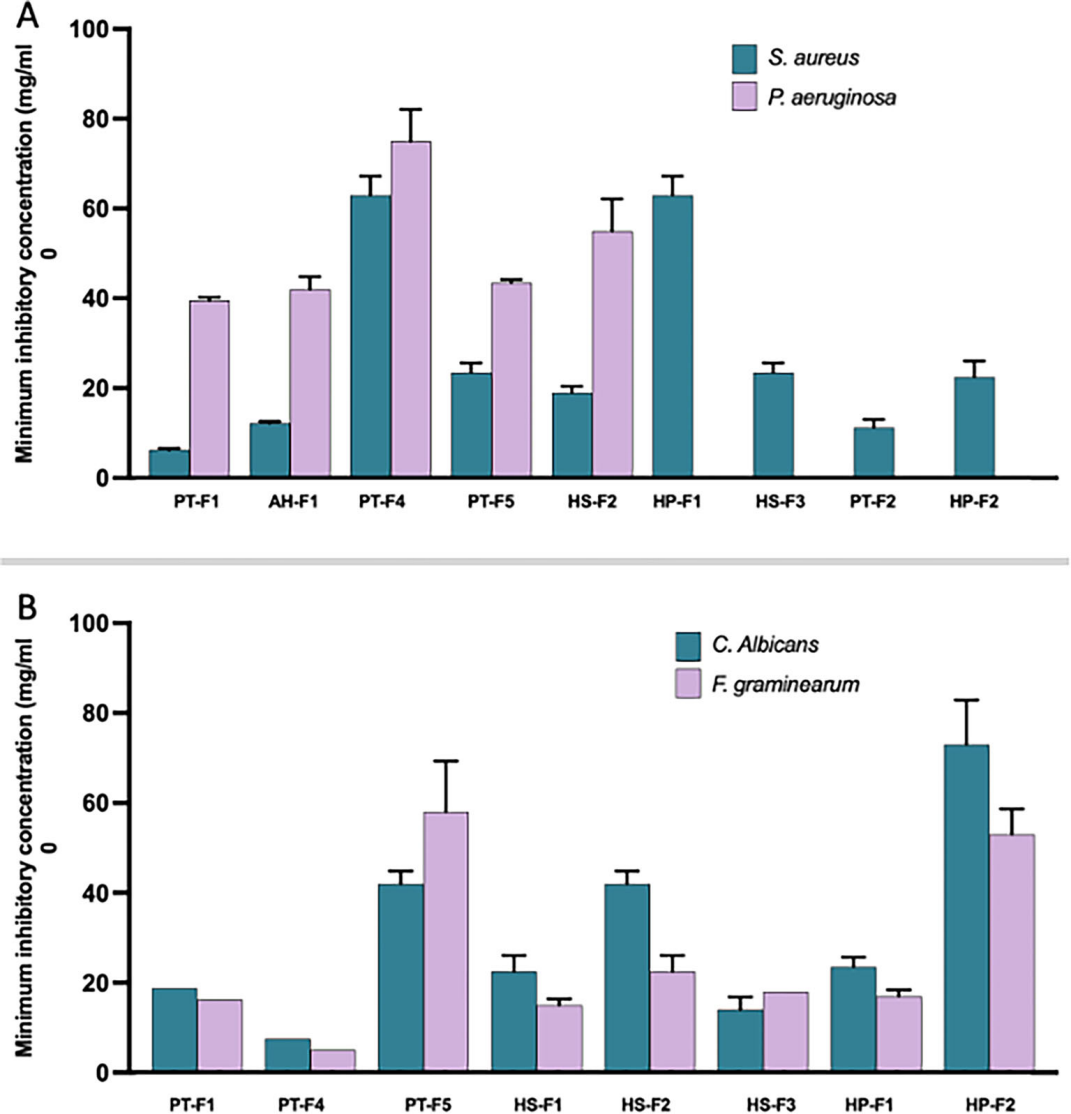
Antimicrobial activities of crude extracts from cultured fungal species, measured as MIC. The graph presents results from two experiments: A) antibacterial activities against Gram-positive indicator *S. aureus* and Gram-negative indicator *P. aeruginosa*, and B) antifungal activities against the human-associated yeast *C. albicans* and the plant pathogenic fungus *F. graminearum.* Data represent the average of three replicates, with bars indicating the standard deviation of the mean.

**Figure 4.**
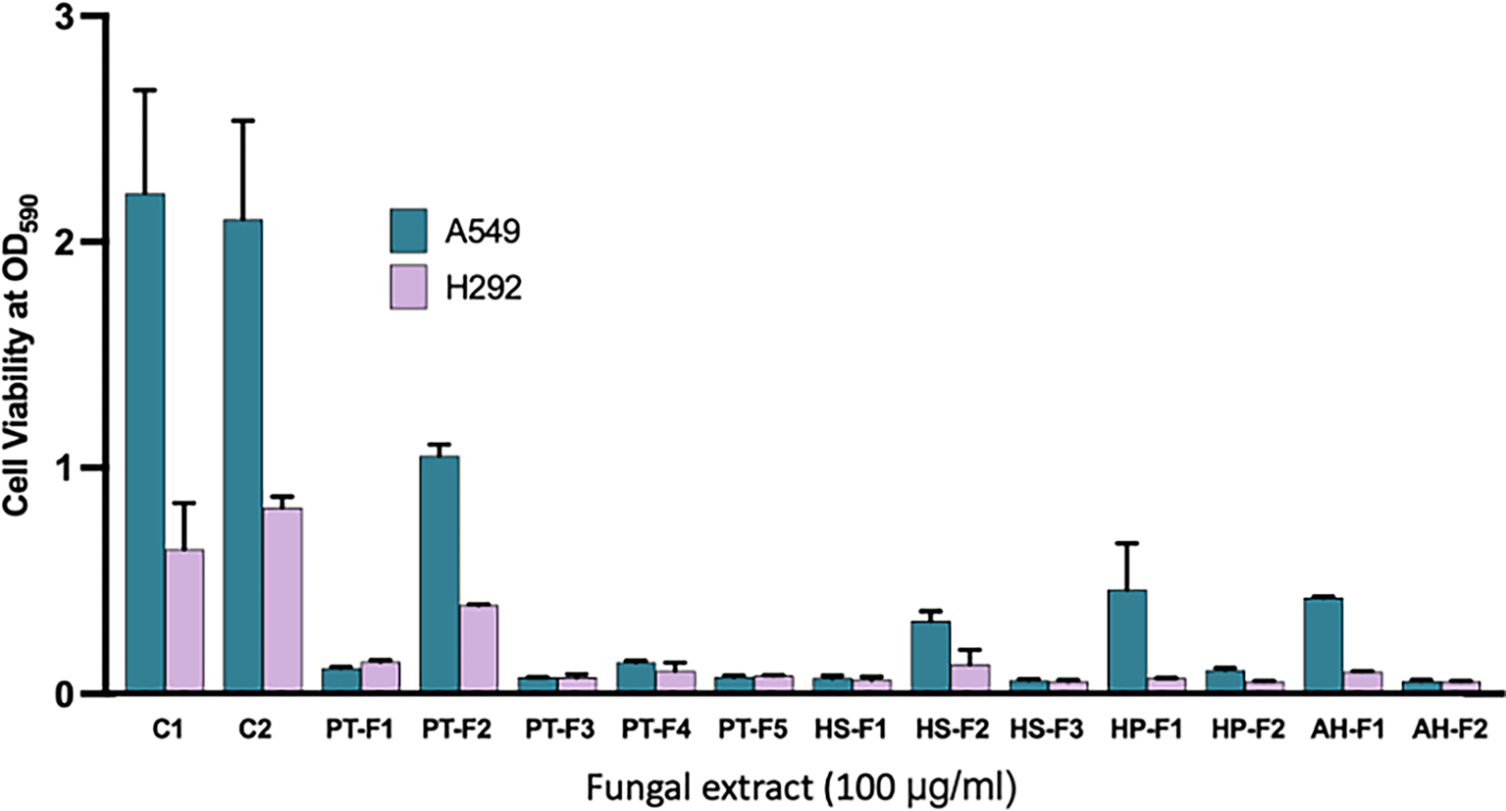
Cytotoxic activities of the fungal extracts against two cancer cell lines (A549 and H292).

To assess the antimicrobial potential of each extract against bacterial and fungal indicators, we conducted two experiments. The first was an agar diffusion test to identify any possible activities of the crude extracts. Extracts that demonstrated positive results based on the diameter of the inhibition zone were subsequently subjected to a broth dilution test to determine their minimum inhibitory concentration (MIC). Data presented in Figure 3A revealed significant antibacterial activity, particularly against the Gram-positive indicator strain *Staphylococcus aureus*, with a total of nine isolates exhibiting positive inhibitory effects. The most potent extracts, characterized by the lowest MIC values, were derived from *Panicum turgidum* (PT-F1, PT-F2) and *Arnebia hispidissima* (AH-F1), followed by *Halocnemum strobilaceum* (HS-F2, HS-F3) and PT-F5, HP-F2. In contrast, activity against *Pseudomonas aeruginosa* was limited, as indicated by the high MIC values for all extracts. The lowest MIC values were recorded for PT-F1 ( *Mucor sp.*), PT-F5 ( *Chaetomium sp.*), and AH-F1 ( *Curvularia sp.*). In terms of antifungal activities, eight fungal extracts showed positive effects against the tested fungal species ( Figure 3B). The most active extracts in inhibiting both *Fusarium graminearum* and *Candida albicans* were PT-F4, followed by PT-F1, HS-F3, HS-F1, and HP-F1. The highest MIC values were observed for PT-F5 ( *Chaetomium sp.*) and HP-F2 ( *Aspergillus sp.*). To assess the preliminary cytotoxic activities of the extracts, we conducted the MTT assay. All extracts demonstrated cytotoxic activity, with the lowest observed for PT-F2 ( *Aspergillus sp.*), as shown in Figure 4.

## Discussion

In this study, we identified 12 fungal species for the first time in the examined desert plants and explored their biochemical characteristics. These fungal isolates belong to eight genera, with *Aspergillus* and *Fusarium* being the most prevalent. Species from the genus *Aspergillus* have been previously isolated from desert plants, such as *Opuntia versicolor* in the Sonoran Desert. ^26^ A study conducted in Saudi Arabia, investigating the cultivable fungal communities in the Arabian Peninsula’s desert soils, also identified *Aspergillus* as the most widespread fungal genus. Similarly, another study identified *Aspergillus* species, specifically *A. niger* and *A. flavus*, among the fungal communities in Saudi Arabia’s Sabkha desert marshes. ^27, 28^ *Aspergillus fumigatus* is known to inhabit many desert plants in arid and semi-arid environments across regions like India, Pakistan, the Mexican desert, and Iran. ^29^ Additionally, *Fusarium* species are dominant as endophytic fungi in various desert plants, including *Cyathea gigantea*, *Calotropis procera*, *Withania somnifera*, and *Aloe vera.* ^30– 33^ *Alternaria species* has been isolated from desert plants in both Jordanian and Saudi Arabian deserts ^34^ and from soil in Al-Kharj, Saudi Arabia. ^35^

### Ecological significance of isolated fungi to desert habitats

Fungi play a crucial role in desert ecosystems by supporting plant health, enhancing nutrient cycling, and providing resilience against environmental stressors. The unique metabolic capabilities of the fungal species identified in this study highlight their importance in these arid environments. Previous research supports the unique metabolic capabilities of these fungal species and highlights their crucial role in promoting plant health. The genus *Aspergillus* is regarded as a prevalent endophytic fungus ^36^ with xerophytic characteristics, allowing it to thrive under the arid conditions and water scarcity typical of desert environments. ^37^ *Aspergillus niger* has been shown to provide a biological shield for host plants by protecting them from pests and pathogens and enhancing their resilience to biotic and abiotic stresses. ^38^ Another fungus identified in this study, *Mucor mucedo* (commonly known as pin mold), is a saprophyte with a broad ecological tolerance and a worldwide distribution. It colonizes decaying organic matter and is capable of rapid growth in environments with limited nutrients. ^39^ *Mucor mucedo* can also survive extreme environmental conditions, such as freezing temperatures, UV radiation, and desiccation. ^40^ Its role in decomposing organic matter yields essential nutrients that support plant development. ^41^ In this study, we also identified *Colletotrichum spaethianum*, a known endophytic fungus. Species within the *Colletotrichum* genus are primarily found in tropical and temperate environments. ^42^ Some *Colletotrichum* species exhibit beneficial activities, such as *C. magna*, which helps plants combat infections caused by *F. oxysporum* and *C. orbiculare.* ^43^ Moreover, *C. gloeosporioides* produces colletotric acid, a potent antifungal compound. ^44^ *Chaetomium globosum*, known as a saprophyte and occasionally an endophyte, has been demonstrated to protect plants against the toxic effects of heavy metals such as copper. ^45^ *Aspergillus terreus*, another species identified in this study, produces metabolites such as phenols, flavonoids, and indole-acetic acid, which stimulate plant growth. Research on tomato plants shows that *A. terreus* enhances shoot and root length, as well as overall chlorophyll content. ^46^ Another study reports that filtrates of *A. terreus*, free from spores and mycelia, significantly reduce spore formation of the plant pathogen *Pythium aphanidermatum*, thereby improving plant growth and protection. ^47^ *Plectosphaerella cucumerina* is another significant fungus identified. Its cell wall contains molecules classified as microbe-associated molecular patterns (MAMPs), which can bind to pattern recognition receptors (PRRs) in *Arabidopsis thaliana*, triggering the plant’s defense mechanisms. ^48^ Additionally, *P. cucumerina* promotes host plant growth by inducing the expression of genes involved in carbohydrate and amino acid synthesis, which enhances the host plant’s growth and can impart similar benefits when transplanted into other plants. ^49, 50^

### Bioactivity of fungal extracts and previous isolated compounds

Our investigation into the crude extracts from various cultured fungal species revealed a rich spectrum of biological activities, including antimicrobial, antioxidant, and anticancer properties. This is supported by previous studies. For instance, *A. niger*, isolated from desert soils in Saudi Arabia, exhibited significant antioxidant activities. ^51^ Furthermore, extracts of *A. niger* and *A. flavus* displayed potent antimicrobial and anticancer activities. ^52^ Additionally, *Aspergillus* species from *Phragmites australis* leaves showed antibacterial effects against *Klebsiella sp.*, *E. coli*, and *S. aureus*, along with antibiofilm activities. ^53^ The extracts also demonstrated cytotoxicity on the breast cancer cell line MCF-7, with an IC50 of 8 μg/μl. Detailed studies have shown that *A. niger* extract can induce cell cycle arrest and apoptosis, ^54^ revealing a composition of diverse hydrocarbons, phthalates, and phenolic derivatives. ^55^ Various metabolites from *A. terreus* have exhibited antibacterial, anticancer, and antioxidant activities. ^56, 57^ Notably, *Asperteramide A* showed potent antibacterial activity against *Klebsiella pneumoniae*, *MRSA*, *Acinetobacter baumannii*, *Enterococcus faecalis*, and *ESBL*-producing *E. coli.* ^58^ Furthermore, *tetracyclic acid A*, isolated from *A. terreus*, has emerged as a valuable anticancer agent by stimulating heat shock responses in tumor cells. ^59^ Crude extracts from *Aspergillus fumigatus*, isolated from mangrove plants in the Sundarbans, demonstrated potent antibacterial activity against both Gram-positive and Gram-negative strains, including *E. coli*, *Micrococcus luteus*, *Pseudomonas aeruginosa*, and *Staphylococcus aureus.* ^60^ Remarkably, an enzyme produced by thermotolerant *A. fumigatus*, known as MGL, exhibited anticancer activity in Hep-G2 and HCT116 cell lines. ^61^ *Fusarium* species isolated from *Cinnamomum kanehirae*, *Selaginella pallescens*, and *Tripterygium wilfordii* displayed antimicrobial activities against *methicillin-resistant S. aureus* and *Candida albicans.* ^62– 65^ A recent study indicated that the ethyl acetate extract of *Fusarium* species possesses the highest antibacterial, antioxidant, and anticancer potential. ^66^

In this study, we identified *Colletotrichum* species with antibacterial and antioxidant activities. Previous studies noted the presence of two *Colletotrichum* sp. in *Andrographis paniculate*, demonstrating their ability to produce antimicrobial and antioxidant compounds. ^67^ Additionally, *Colletotrichum acutatum*, isolated from *Angelica sinensis*, produces molecules exhibiting antimalarial, antioxidant, antibacterial, anti-proliferative, and antibiofilm activity. ^68^ Extracts from *Colletotrichum* sp. CG1-7, isolated from *Arrabidaea chica*, displayed antioxidant activity comparable to quercetin. ^69^ Furthermore, two metabolites derived from *Colletotrichum* sp., phthalide and isocoumarins, have shown potential as antioxidant agents and in inhibiting cancer cell growth in the HepG2 cell line. ^70^ Another compound, palmitoylethanolamide (PEA), isolated from *C. gloeosporioides*, demonstrated potential anticancer activity against human breast cancer cells via apoptosis induction. ^71^ Interestingly, the production of valuable antimicrobial and antioxidant compounds from various *Colletotrichum* species is significantly enhanced by light spectrum treatment. ^72^ Additionally, we reported on the activities of extracts from *Alternaria* species, including potent antifungal and anticancer activities. Previous data show that extracts of *A. alternata* exhibit antibacterial activity. ^73^ Compounds isolated from *A. alternata* include alternariol, tenuazonic acid, levofuraltadone, and kigelinone, which have antibacterial and/or anticancer activities. ^74, 75^ Another fungus identified in our study is *Chaetomium*, exhibiting antioxidant and anticancer activities. Bioactive metabolites from *Chaetomium globosum* have been reported, displaying anticancer, antimicrobial, anti-inflammatory, antiviral, and antioxidant activities. ^76, 77^ Notably, chrysophanol alkaloid from *Chaetomium* shows anticancer activities against multiple cancer types, along with its antimicrobial properties. ^78– 81^ Another compound, Chetomin, produced by the *Chaetomium* genus, has been noted for its ability to block hypoxic-inducible transcription, thereby suppressing tumor growth. ^82^ We also identified *Plectosphaerella* species with antifungal, anticancer and antioxidant activities. Previous reports indicate that *P. cucumerina* extracts exhibit antibiofilm and anti-virulence activity against *Pseudomonas aeruginosa*, likely due to the presence of emodin and patulin compounds. ^83^ Moreover, studies have shown the potential of *P. cucumerina* in providing protection against nematodes. ^84, 85^

Lastly, we reported activities of *Curvularia* species, including antibacterial activities against both Gram positive and negative pathogens. Previous research indicates that extracts from *C. lunata* possess antimicrobial and antioxidant activities, with low cytotoxic effects against the ATCC-CCL-81 cell line. ^86^ Furthermore, *Curvularia* species are known for producing metabolites with antibacterial, antioxidant, and anticancer activities. For instance, *Curvularia* sp. G6-32, isolated from *Sapindus saponaria*, generates epoxyquinone, noted for its antioxidant potential. ^87^ Regarding anticancer properties, *Curvularia australiensis* FC2AP, isolated from *Aegle marmelos* leaves, produces flavonoids that exhibit anti-cervical cancer and anti-inflammatory effects. ^88^ Additionally, *Curvularia* sp. from *Terminalia laxiflora* generates bioactive peptides, shown to suppress tumor growth and inhibit angiogenesis. ^89^

### Desert microbiomes: Implications for climate change solutions

Desert fungi hold significant potential to mitigate climate change impacts due to their unique adaptations to extreme heat, drought, and salinity. *Colletotrichum* species, for instance, serve as eco-friendly protectors against abiotic drought stress, which can severely damage plants and crops. *Colletotrichum alatae* secretes a heteropolysaccharide rich in β-glucan, enhancing drought resilience and optimizing rice cultivation in severely drought-affected areas. ^90^ Furthermore, *Colletotrichum* species have been identified as drought-tolerant fungi, significantly contributing to plant growth in arid environments. ^91^ *Chaetomium globosum* is recognized for its salt tolerance, promoting plant resilience in saline conditions. ^92^ Reports indicate that *Chaetomium globosum* can enhance the survival and growth of salt-sensitive crops under drought stress. ^93^ Other fungi, such as *Aspergillus fumigatus*, thrive at extreme temperatures and demonstrate potential in protecting sensitive plants like wheat from drought. ^94^ Research has also highlighted the ability of *C. lunata* inoculum to improve resistance to salt and drought in rice, thus enhancing overall plant growth. ^95^

Desert fungi exhibit various environmental applications. For example, *A. niger* produces novel xylose transporters that efficiently convert lignocellulosic biomass into eco-friendly biofuels. ^96^ *Mucor mucedo* is increasingly recognized for its ability to degrade hydrocarbons. A study found that immobilizing *M. mucedo* on corncob particles enhanced its efficacy in remediating pyrene-contaminated agricultural soil. ^97^ Further investigation into *M. mucedo* revealed that exopolymer substances (EPS) play a crucial role in degrading polycyclic aromatic hydrocarbons, suggesting its application in environmental cleanup efforts. ^98^ Additionally, *C. lunata* has been shown to enhance bioremediation of hydrocarbon-contaminated soil in conjunction with the plant *Luffa aegyptiaca*, facilitating the degradation of accumulated hydrocarbons. ^99^ The *Plectosphaerella cucumerina* AR1 strain has also demonstrated the ability to degrade nicosulfuron, an herbicide commonly applied to maize crops, which contributes to groundwater and surface stream contamination. ^100^

## Conclusion

The Arabian Peninsula desert is home to diverse microbial communities that offer significant applications in drug discovery, as well as industrial and environmental interventions related to bioremediation and climate change solutions. Given the unique adaptations of these microorganisms, a comprehensive profiling of the desert microbiome, encompassing both bacterial and fungal communities, should be a research priority. This effort will not only enhance our understanding of desert ecosystems but also unlock the potential of these microbes for sustainable applications.

## Authors contribution

WM designed the study, collected the plants, isolated fungi, performed DNA extraction, PCR experiments, performed large scale fermentation and extraction, conducted biological assays, performed statistical and data analysis, designed and developed figures, and wrote the manuscript. RG conducted cytotoxicity experiment. NA contributed to isolation and purification of individual isolates, antimicrobial assays, data analysis and discussion. TAI designed the study, experimental protocols and analyzed data. All authors reviewed, edited, and approved the final version of the manuscript.

## Ethics and consent

Ethical approval and consent were not required.

## Data Availability

Figshare: “Biological Activities of Fungal Isolates from Arabian Desert.” DOI: 10.6084/m9.figshare.27925029. ^101^ This project contains the following underlying data:
• antimicrobial assay results, antioxidant activity data, and anticancer activity data. Data are available under the terms of the Creative Commons Attribution 4.0 International license (CC-BY 4.0). All sequencing data generated form this study have been made publicly available and have been deposited into GenBank. Links to access data are as follows; GenBank: *Mucor* sp. PQ480174; https://www.ncbi.nlm.nih.gov/search/all/?term=PQ480174 GenBank: *Aspergillus* sp. PQ480175; https://www.ncbi.nlm.nih.gov/search/all/?term=PQ480175 GenBank: *Colletotricum* sp. PQ480176; https://www.ncbi.nlm.nih.gov/search/all/?term=PQ480176 GenBank: *Alternaria* sp. PQ480177; https://www.ncbi.nlm.nih.gov/search/all/?term=PQ480177 GenBank: *Chaetomium* sp. PQ480178; https://www.ncbi.nlm.nih.gov/search/all/?term=PQ480178 GenBank: *Aspergillus* sp. PQ480179; https://www.ncbi.nlm.nih.gov/search/all/?term=PQ480179 GenBank: *Aspergillus* sp. PQ480180; https://www.ncbi.nlm.nih.gov/search/all/?term=PQ480180 GenBank: *Fusarium* sp. PQ480181; https://www.ncbi.nlm.nih.gov/search/all/?term=PQ480181 GenBank: *Plecotosphaerella* sp. PQ480182; https://www.ncbi.nlm.nih.gov/search/all/?term=PQ480182 GenBank: *Aspergillus* sp. PQ480183; https://www.ncbi.nlm.nih.gov/search/all/?term=PQ480183 GenBank: *Curvularia* sp. PQ480184; https://www.ncbi.nlm.nih.gov/search/all/?term=PQ480184 GenBank: *Fusarium* sp. PQ480185; https://www.ncbi.nlm.nih.gov/search/all/?term=PQ480185
