# Supplementary material for: Revealing culturable fungal microbiome communities from the Arabian Peninsula desert representing a unique source of biochemicals for drug discovery and biotechnology
Source: F1000Res. 2024 Dec 17;13:1527. [Version 1] doi: 10.12688/f1000research.158130.1 (PMC11914873; doi:10.12688/f1000research.158130.1)
Supplement: Peer review history

# Reviewer report: Reviewer response for version 1

**DOI:** 10.5256/f1000research.173683.r370439

**Published:** 2025-03-17

**Authors:** Hanan S Anbar

**Affiliations:** 1 Dubai Pharmacy College for Girls, Dubai, United Arab Emirates

The manuscript titled "Revealing culturable fungal microbiome communities from the Arabian Peninsula desert representing a unique source of biochemicals for drug discovery and biotechnology" is well-structured and presents an interesting investigation into Natural products discovery from the fungal microbiome of desert plants. The research highlights the biotechnological potential of extremophilic fungi, making a valuable contribution to drug discovery and biotechnology . The topic is also crucial to climate change research by identifying novel   fungal species that supports the growth of desert plants under the harsh environmental conditions of the desert and could be further studied to support the growth of climate sensitive ecosystems .

 -The abstract is informative and clearly demonstrates the research question, gap, findings, and impact of the research.

 - The introduction effectively presents the study's rationale  and gives a comprehensive background required to better understand the context and research question. 

 - The experiments are designed based on standard protocols and each experiment contains sufficient numbers of replicates and appropriate positive and negative controls. 

 - The data tables contains statistical analysis supporting the significance of the results however this also could be beneficial to the reader if reflected in the figures to be more obvious 

 - The discussion includes a comparative analysis of their findings with similar studies from other desert ecosystems which helps to contextualize the significance of the results.

 Overall the research fills a significant gap enriching natural products discovery from extreme sources represented here in desert fungi and could potentially lead to discovery of novel natural products.

Is the work clearly and accurately presented and does it cite the current literature?

Yes

If applicable, is the statistical analysis and its interpretation appropriate?

Yes

Are all the source data underlying the results available to ensure full reproducibility?

Yes

Is the study design appropriate and is the work technically sound?

Yes

Are the conclusions drawn adequately supported by the results?

Yes

Are sufficient details of methods and analysis provided to allow replication by others?

Yes

Reviewer Expertise:

Pharmacology, Toxicology, Molecular Biology

I confirm that I have read this submission and believe that I have an appropriate level of expertise to confirm that it is of an acceptable scientific standard.

# Reviewer report: Reviewer response for version 1

**DOI:** 10.5256/f1000research.173683.r357007

**Published:** 2025-02-04

**Authors:** Sotirios Pilafidis

**Affiliations:** 1 University of the Aegean, Myrina, Lemnos, Greece; 2 Research and Development, OPUS CACTUS, Groningen, The Netherlands

The article presents novel work being done in previously understudies desert regions. The authors have identified and selected important key plant species of the region to isolate fungi from. The authors used solid standard practices to isolate and characterize the isolated species. Molecular identification by whole genome sequencing is highly recommended in the future. Overall the article is a significant contribution to the understudied knowledge base of arid regions microbiome diversity.

Is the work clearly and accurately presented and does it cite the current literature?

Yes

If applicable, is the statistical analysis and its interpretation appropriate?

Yes

Are all the source data underlying the results available to ensure full reproducibility?

Yes

Is the study design appropriate and is the work technically sound?

Yes

Are the conclusions drawn adequately supported by the results?

Yes

Are sufficient details of methods and analysis provided to allow replication by others?

Yes

Reviewer Expertise:

Microbiology, Bioconversion, Biotechnology, Sustainability, Arid regions biodiversity.

I confirm that I have read this submission and believe that I have an appropriate level of expertise to confirm that it is of an acceptable scientific standard.
